# Supplementary material for: An intriguing characteristic of enhancer-promoter interactions
Source: BMC Genomics. 2021 Mar 8;22:163. doi: 10.1186/s12864-021-07440-5 (PMC7938488; doi:10.1186/s12864-021-07440-5)
Supplement: Supplementary file 1 — Additional file 1 Table S2. BCC statistics for promoters. Table S3. Clusters of enhancers. An enhancer shares at least one promoter target with all the other enhancers in its cluster. Table S4. The distance between each consecutive enhancer pairs in an enhancer clusters are shown in the left columns of the table. The right columns show the distance between each consecutive target pairs of the targets of the enhancers in an enhancer cluster. Table S5. The average distance between each enhancer pairs in enhancer clusters and in randomly chosen enhancers that do not belong to any cluster. Table S6. Overlap between enhancer clusters and super-enhancers. Table S7. Average percentage of enhancers in the same clusters mapped in a common TAD or TAD gap. On average, almost all of the enhancers in a cluster were found to be located within the same TAD or TAD gap. Table S8. Percentages of common enhancer clusters between two cell lines. Table S9. Gene ontology enrichment analysis for the target genes of the enhancer clusters. Table S10. The average sequence similarity score among the enhancers in a cluster and in randomly chosen enhancers that do not belong to any cluster. [file 12864_2021_7440_MOESM1_ESM.docx]

**An intriguing characteristic of enhancer-promoter interactions**

Table S1: BCC statistics for enhancers. The BCC of the enhancers in the real IEPs are shown for different samples. The BCC of the enhancers in random IEPs are also shown along with the p-values of the nonparametric statistical test supporting the difference between the BCC values in real and random IEPs. All the statistics are shown for both “all” enhancers and the enhancers interacting with “multiple” promoters.

| Experiments | Cell lines | IEPs | Enhancers | BCC of enhancers | | BCC of enhancers in random IEPs with p-values in parenthesis | |
| --- | --- | --- | --- | --- | --- | --- | --- |
|  |  |  |  | All | Multiple | All | Multiple |
| FANTOM Gencode Rao looplist | GM12878 | 294 | 229 | 0.97 | 0.96 | 0.51 (0) | 0.34 (0) |
|  | HELA | 11 | 10 | 1 | 0 | 0 (0) | 0 (NA) |
|  | HMEC | 260 | 201 | 0.97 | 0.96 | 0.37 (0) | 0.17 (0) |
|  | HUVEC | 9 | 9 | 1 | 0 | 0 (0) | 0 (NA) |
|  | IMR90 | 144 | 125 | 1 | 1 | 0.33 (0) | 0.2 (0) |
|  | K562 | 47 | 37 | 1 | 1 | 0 (0) | 0 (0) |
|  | KBM7 | 8 | 5 | 0 | 0 | 0 (NA) | 0 (NA) |
|  | NHEK | 0 | 0 | NA | NA | NA (NA) | NA (NA) |
| FANTOM Gencode Rao cutoff 400 | GM12878 | 902 | 783 | 0.97 | 0.85 | 0.78 (0) | 0.38 (0) |
| FANTOM Gencode Rao cutoff 300 | GM12878 | 1138 | 974 | 0.95 | 0.82 | 0.76 (0) | 0.39 (0) |
| FANTOM Gencode Rao cutoff 200 | GM12878 | 2695 | 2091 | 0.9 | 0.74 | 0.62 (0) | 0.36 (0) |
| FANTOM Gencode Rao cutoff 150 | GM12878 | 4184 | 3002 | 0.88 | 0.74 | 0.56 (0) | 0.34 (0) |
| FANTOM Gencode Rao cutoff 100 | GM12878 | 7527 | 4488 | 0.81 | 0.7 | 0.43 (0) | 0.28 (0) |
|  | HMEC | 313 | 277 | 0.93 | 0.67 | 0.53 (0) | 0.07 (0) |
|  | HUVEC | 43 | 41 | 0.92 | 0.5 | 0 (0) | 0 (NA) |
|  | IMR90 | 525 | 468 | 0.96 | 0.72 | 0.83 (0) | 0.42 (0) |
|  | K562 | 506 | 440 | 0.96 | 0.83 | 0.8 (0) | 0.39 (0) |
|  | KBM7 | 1465 | 1308 | 0.94 | 0.7 | 0.84 (0) | 0.43 (0) |
|  | NHEK | 211 | 200 | 0.95 | 0.5 | 0.49 (0) | 0.3 (NA) |
| FANTOM Gencode Rao cutoff 50 | GM12878 | 19623 | 7599 | 0.73 | 0.66 | 0.25 (0) | 0.19 (0) |
|  | HMEC | 854 | 702 | 0.94 | 0.85 | 0.68 (0) | 0.4 (0) |
|  | HUVEC | 254 | 237 | 0.95 | 0.81 | 0.58 (0) | 0.1 (0) |
|  | IMR90 | 1643 | 1319 | 0.91 | 0.75 | 0.66 (0) | 0.39 (0) |
|  | K562 | 1734 | 1368 | 0.89 | 0.73 | 0.64 (0) | 0.39 (0) |
|  | KBM7 | 4033 | 3274 | 0.9 | 0.74 | 0.7 (0) | 0.37 (0) |
|  | NHEK | 462 | 407 | 0.92 | 0.69 | 0.78 (0) | 0.4 (0) |
| FANTOM Gencode Rao cutoff 30 | GM12878 | 29348 | 8670 | 0.71 | 0.65 | 0.48 (0) | 0.47 (0) |
|  | HMEC | 1786 | 1451 | 0.92 | 0.78 | 0.83 (0) | 0.53 (0) |
|  | HUVEC | 582 | 518 | 0.95 | 0.81 | 0.9 (0) | 0.52 (0) |
|  | IMR90 | 3077 | 2235 | 0.87 | 0.73 | 0.76 (0) | 0.54 (0) |
|  | K562 | 2872 | 2021 | 0.85 | 0.71 | 0.74 (0) | 0.52 (0) |
|  | KBM7 | 7047 | 5564 | 0.88 | 0.72 | 0.81 (0) | 0.52 (0) |
|  | NHEK | 1011 | 885 | 0.93 | 0.76 | 0.88 (0) | 0.52 (0) |
| ChromHMM Gencode Rao looplist | GM12878 | 2384 | 1914 | 0.99 | 0.96 | 0.67 (0) | 0.39 (0) |
|  | HELA | 37 | 32 | 1 | 1 | 0.1 (0) | 0.1 (0) |
|  | HMEC | 2558 | 1907 | 0.99 | 0.98 | 0.59 (0) | 0.36 (0) |
|  | HUVEC | 95 | 86 | 1 | 1 | 0.22 (0) | 0.17 (0) |
|  | IMR90 | 554 | 490 | 1 | 0.99 | 0.77 (0) | 0.45 (0) |
|  | K562 | 638 | 536 | 1 | 1 | 0.74 (0) | 0.44 (0) |
|  | NHEK | 0 | 0 | NA | NA | NA (NA) | NA (NA) |
| ChromHMM Gencode Rao cutoff 400 | GM12878 | 11097 | 9343 | 0.93 | 0.78 | 0.75 (6.72E-12) | 0.42 (0) |
| ChromHMM Gencode Rao cutoff 300 | GM12878 | 14846 | 12347 | 0.92 | 0.78 | 0.74 (1.52E-11) | 0.42 (0) |
| ChromHMM Gencode Rao cutoff 200 | GM12878 | 33072 | 24664 | 0.81 | 0.67 | 0.64 (1.17E-11) | 0.37 (0) |
| ChromHMM Gencode Rao cutoff 150 | GM12878 | 51174 | 34925 | 0.8 | 0.67 | 0.57 (0) | 0.34 (0) |
| ChromHMM Gencode Rao cutoff 100 | GM12878 | 89712 | 51676 | 0.74 | 0.64 | 0.46 (0) | 0.29 (0) |
|  | HMEC | 4081 | 3635 | 0.94 | 0.76 | 0.81 (0) | 0.41 (0) |
|  | HUVEC | 499 | 458 | 0.98 | 0.86 | 0.85 (0) | 0.48 (0) |
|  | IMR90 | 2415 | 2118 | 0.97 | 0.88 | 0.78 (0) | 0.41 (0) |
|  | K562 | 8062 | 6835 | 0.93 | 0.76 | 0.75 (0) | 0.42 (0) |
|  | NHEK | 3291 | 3028 | 0.96 | 0.75 | 0.86 (0) | 0.44 (0) |
| ChromHMM Gencode Rao cutoff 50 | GM12878 | 231522 | 88850 | 0.64 | 0.6 | 0.27 (0) | 0.19 (0) |
|  | HMEC | 11191 | 9131 | 0.92 | 0.78 | 0.69 (0) | 0.39 (0) |
|  | HUVEC | 3396 | 3073 | 0.96 | 0.8 | 0.83 (0) | 0.44 (0) |
|  | IMR90 | 7270 | 5765 | 0.93 | 0.79 | 0.67 (1.73E-12) | 0.39 (0) |
|  | K562 | 28590 | 21084 | 0.86 | 0.7 | 0.63 (0) | 0.37 (0) |
|  | NHEK | 7017 | 6103 | 0.94 | 0.77 | 0.78 (0) | 0.43 (0) |
| Jin | IMR90 | 50800 | 44239 | 0.94 | 0.79 | 0.81 (0) | 0.44 (0) |
| FANTOM Gencode Jin | IMR90 | 1167 | 743 | 0.9 | 0.84 | 0.51 (0) | 0.33 (0) |
| ChromHMM Gencode Jin | IMR90 | 5303 | 3383 | 0.93 | 0.87 | 0.53 (0) | 0.32 (0) |
| FANTOM Gencode Chiapet | K562 | 2916 | 1585 | 0.8 | 0.75 | 0.41 (0) | 0.28 (0) |
|  | MCF7 | 2190 | 1471 | 0.89 | 0.83 | 0.55 (0) | 0.35 (0) |
| ChromHMM Gencode Chiapet | K562 | 33449 | 19550 | 0.86 | 0.78 | 0.46 (1.74E-11) | 0.3 (0) |
| FANTOM Gencode Javierre | Ery | 74 | 44 | 1 | 1 | 0.41 (0) | 0.33 (0) |
|  | Mac0 | 88 | 59 | 0.98 | 0.94 | 0.51 (0) | 0.37 (0) |
|  | Mac1 | 215 | 144 | 1 | 1 | 0.54 (0) | 0.37 (0) |
|  | Mac2 | 112 | 75 | 0.99 | 0.96 | 0.53 (0) | 0.34 (0) |
|  | MK | 100 | 65 | 0.96 | 0.9 | 0.52 (0) | 0.34 (0) |
|  | Mon | 139 | 82 | 1 | 1 | 0.43 (0) | 0.32 (0) |
|  | nCD4 | 86 | 58 | 1 | 1 | 0.52 (0) | 0.35 (0) |
|  | nCD8 | 84 | 55 | 1 | 1 | 0.5 (0) | 0.36 (0) |
|  | Neu | 178 | 109 | 1 | 1 | 0.45 (0) | 0.32 (0) |
| ChromHMM Gencode Javierre | Ery | 4484 | 2471 | 0.98 | 0.98 | 0.42 (0) | 0.3 (0) |
|  | Mac0 | 2003 | 1097 | 0.99 | 0.99 | 0.41 (0) | 0.29 (0) |
|  | Mac1 | 4867 | 2996 | 0.97 | 0.96 | 0.49 (0) | 0.33 (0) |
|  | Mac2 | 3733 | 2298 | 0.99 | 0.99 | 0.49 (0) | 0.33 (0) |
|  | MK | 2629 | 1744 | 0.99 | 0.98 | 0.55 (0) | 0.35 (0) |
|  | Mon | 2483 | 1547 | 0.96 | 0.94 | 0.49 (0) | 0.34 (0) |
|  | nCD4 | 2975 | 1546 | 0.99 | 0.99 | 0.39 (0) | 0.28 (0) |
|  | nCD8 | 2774 | 1623 | 0.98 | 0.97 | 0.46 (0) | 0.31 (0) |
|  | Neu | 4661 | 2739 | 0.99 | 0.98 | 0.46 (0) | 0.32 (0) |
| FANTOM Gencode SPRITE cutoff 100 | GM12878 | 38 | 28 | 1 | 1 | 0.2 (0) | 0 (0) |
| FANTOM Gencode SPRITE cutoff 50 | GM12878 | 497 | 317 | 0.92 | 0.8 | 0.46 (0) | 0.35 (0) |
| FANTOM Gencode SPRITE cutoff 30 | GM12878 | 3381 | 2151 | 0.92 | 0.84 | 0.45 (0) | 0.3 (0) |
| ChromHMM Gencode SPRITE cutoff 100 | GM12878 | 622 | 453 | 0.99 | 0.97 | 0.56 (0) | 0.3 (0) |
| ChromHMM Gencode SPRITE cutoff 50 | GM12878 | 4794 | 3213 | 0.95 | 0.89 | 0.5 (0) | 0.32 (0) |
| ChromHMM Gencode SPRITE cutoff 30 | GM12878 | 36027 | 21870 | 0.9 | 0.81 | 0.48 (1.22E-11) | 0.3 (0) |

Table S2: BCC statistics for promoters. The BCC of the promoters in real IEPs are shown for different samples. The BCC of the promoters in random IEPs are also shown along with the p-values of the nonparametric statistical test supporting the difference between the BCC values in real and random IEPs. All the statistics are shown for both “all” promoters and the promoters interacting with “multiple” enhancers.

| Experiments | Cell lines | IEPs | Promoters | BCC of Promoters | | BCC of promoters in random IEPs with p-values in parenthesis | |
| --- | --- | --- | --- | --- | --- | --- | --- |
|  |  |  |  | All | Multiple | All | Multiple |
| FANTOM Gencode Rao looplist | GM12878 | 294 | 186 | 0.97 | 0.95 | 0.52 (0) | 0.28 (0) |
|  | HELA | 11 | 8 | 1 | 0 | 0 (0) | 0 (NA) |
|  | HMEC | 260 | 179 | 0.96 | 0.91 | 0.52 (0) | 0.27 (0) |
|  | HUVEC | 9 | 6 | 0 | 0 | 0 (NA) | 0 (NA) |
|  | IMR90 | 144 | 112 | 1 | 1 | 0.48 (0) | 0.08 (0) |
|  | K562 | 47 | 39 | 1 | 1 | 0.1 (0) | 0.1 (0) |
|  | KBM7 | 8 | 8 | 1 | 0 | 0 (0) | 0 (NA) |
|  | NHEK | 0 | 0 | NA | NA | NA (NA) | NA (NA) |
| FANTOM Gencode Rao cutoff 400 | GM12878 | 902 | 683 | 0.95 | 0.81 | 0.62 (0) | 0.37 (0) |
| FANTOM Gencode Rao cutoff 300 | GM12878 | 1138 | 848 | 0.92 | 0.78 | 0.57 (0) | 0.33 (0) |
| FANTOM Gencode Rao cutoff 200 | GM12878 | 2695 | 1663 | 0.83 | 0.7 | 0.43 (0) | 0.29 (0) |
| FANTOM Gencode Rao cutoff 150 | GM12878 | 4184 | 2292 | 0.81 | 0.7 | 0.38 (0) | 0.25 (0) |
| FANTOM Gencode Rao cutoff 100 | GM12878 | 7527 | 3475 | 0.76 | 0.66 | 0.32 (0) | 0.21 (0) |
|  | HMEC | 313 | 288 | 0.95 | 0.7 | 0.94 (0) | 0.17 (0) |
|  | HUVEC | 43 | 36 | 0.75 | 0.5 | 0 (0) | 0 (NA) |
|  | IMR90 | 525 | 438 | 0.93 | 0.71 | 0.7 (0) | 0.39 (0) |
|  | K562 | 506 | 404 | 0.92 | 0.81 | 0.68 (0) | 0.39 (0) |
|  | KBM7 | 1465 | 1285 | 0.92 | 0.71 | 0.79 (0) | 0.42 (0) |
|  | NHEK | 211 | 190 | 0.91 | 0.5 | 0.72 (0) | 0.2 (NA) |
| FANTOM Gencode Rao cutoff 50 | GM12878 | 19623 | 6631 | 0.69 | 0.62 | 0.23 (0) | 0.16 (0) |
|  | HMEC | 854 | 719 | 0.95 | 0.84 | 0.75 (0) | 0.38 (0) |
|  | HUVEC | 254 | 211 | 0.84 | 0.63 | 0.53 (0) | 0.21 (0) |
|  | IMR90 | 1643 | 1232 | 0.88 | 0.75 | 0.62 (0) | 0.35 (0) |
|  | K562 | 1734 | 1218 | 0.85 | 0.72 | 0.54 (0) | 0.32 (0) |
|  | KBM7 | 4033 | 3209 | 0.89 | 0.73 | 0.65 (0) | 0.38 (0) |
|  | NHEK | 462 | 386 | 0.89 | 0.67 | 0.73 (0) | 0.41 (0) |
| FANTOM Gencode Rao cutoff 30 | GM12878 | 29348 | 8320 | 0.66 | 0.61 | 0.48 (0) | 0.45 (0) |
|  | HMEC | 1786 | 1441 | 0.92 | 0.76 | 0.83 (0) | 0.49 (0) |
|  | HUVEC | 582 | 457 | 0.91 | 0.76 | 0.81 (0) | 0.49 (0) |
|  | IMR90 | 3077 | 2050 | 0.85 | 0.72 | 0.73 (0) | 0.52 (0) |
|  | K562 | 2872 | 1815 | 0.82 | 0.69 | 0.7 (0) | 0.49 (0) |
|  | KBM7 | 7047 | 5304 | 0.86 | 0.69 | 0.79 (0) | 0.49 (0) |
|  | NHEK | 1011 | 802 | 0.88 | 0.75 | 0.81 (0) | 0.51 (0) |
| ChromHMM Gencode Rao looplist | GM12878 | 2384 | 674 | 0.95 | 0.93 | 0.13 (0) | 0.12 (0) |
|  | HELA | 37 | 17 | 1 | 1 | 0 (0) | 0 (0) |
|  | HMEC | 2558 | 735 | 0.97 | 0.96 | 0.15 (0) | 0.14 (0) |
|  | HUVEC | 95 | 31 | 1 | 1 | 0 (0) | 0 (0) |
|  | IMR90 | 554 | 310 | 0.99 | 0.98 | 0.51 (0) | 0.24 (0) |
|  | K562 | 638 | 197 | 1 | 1 | 0.12 (0) | 0.12 (0) |
|  | NHEK | 0 | 0 | NA | NA | NA (NA) | 0 (NA) |
| ChromHMM Gencode Rao cutoff 400 | GM12878 | 11097 | 3899 | 0.66 | 0.62 | 0.18 (0) | 0.15 (0) |
| ChromHMM Gencode Rao cutoff 300 | GM12878 | 14846 | 4777 | 0.65 | 0.61 | 0.16 (0) | 0.14 (0) |
| ChromHMM Gencode Rao cutoff 200 | GM12878 | 33072 | 7412 | 0.57 | 0.53 | 0.11 (0) | 0.1 (0) |
| ChromHMM Gencode Rao cutoff 150 | GM12878 | 51174 | 8688 | 0.56 | 0.53 | 0.09 (0) | 0.08 (0) |
| ChromHMM Gencode Rao cutoff 100 | GM12878 | 89712 | 10080 | 0.54 | 0.52 | 0.06 (0) | 0.05 (0) |
|  | HMEC | 4081 | 2410 | 0.74 | 0.66 | 0.4 (0) | 0.28 (0) |
|  | HUVEC | 499 | 283 | 0.84 | 0.79 | 0.26 (0) | 0.18 (0) |
|  | IMR90 | 2415 | 1418 | 0.91 | 0.84 | 0.41 (0) | 0.29 (0) |
|  | K562 | 8062 | 3005 | 0.64 | 0.59 | 0.19 (0) | 0.16 (0) |
|  | NHEK | 3291 | 1784 | 0.71 | 0.64 | 0.35 (0) | 0.26 (0) |
| ChromHMM Gencode Rao cutoff 50 | GM12878 | 231522 | 12998 | 0.49 | 0.48 | 0.03 (0) | 0.03 (0) |
|  | HMEC | 11191 | 5169 | 0.71 | 0.65 | 0.26 (0) | 0.21 (0) |
|  | HUVEC | 3396 | 1660 | 0.7 | 0.65 | 0.27 (0) | 0.21 (0) |
|  | IMR90 | 7270 | 3540 | 0.81 | 0.73 | 0.29 (0) | 0.22 (0) |
|  | K562 | 28590 | 6604 | 0.55 | 0.52 | 0.12 (0) | 0.11 (0) |
|  | NHEK | 7017 | 2851 | 0.69 | 0.64 | 0.22 (0) | 0.18 (0) |
| Jin | IMR90 | 50800 | 8117 | 0.11 | 0.11 | 0.09 (0) | 0.08 (0) |
| FANTOM Gencode Jin | IMR90 | 1167 | 401 | 0.77 | 0.73 | 0.23 (0) | 0.17 (0) |
| ChromHMM Gencode Jin | IMR90 | 5303 | 617 | 0.68 | 0.66 | 0.07 (0) | 0.06 (0) |
| FANTOM Gencode Chiapet | K562 | 2916 | 1869 | 0.86 | 0.75 | 0.52 (0) | 0.31 (0) |
|  | MCF7 | 2190 | 1195 | 0.86 | 0.75 | 0.43 (0) | 0.25 (0) |
| ChromHMM Gencode Chiapet | K562 | 33449 | 6439 | 0.67 | 0.65 | 0.11 (0) | 0.1 (0) |
| FANTOM Gencode Javierre | Ery | 74 | 64 | 1 | 1 | 0.79 (0) | 0.44 (0) |
|  | Mac0 | 88 | 64 | 0.98 | 0.95 | 0.59 (0) | 0.36 (0) |
|  | Mac1 | 215 | 153 | 1 | 1 | 0.6 (0) | 0.35 (0) |
|  | Mac2 | 112 | 85 | 0.98 | 0.96 | 0.64 (0) | 0.38 (0) |
|  | MK | 100 | 81 | 0.98 | 0.89 | 0.73 (0) | 0.38 (0) |
|  | Mon | 139 | 94 | 1 | 1 | 0.57 (0) | 0.31 (0) |
|  | nCD4 | 86 | 64 | 1 | 1 | 0.63 (0) | 0.39 (0) |
|  | nCD8 | 84 | 67 | 1 | 1 | 0.68 (0) | 0.42 (0) |
|  | Neu | 178 | 137 | 1 | 1 | 0.66 (0) | 0.39 (0) |
| ChromHMM Gencode Javierre | Ery | 4484 | 539 | 0.93 | 0.92 | 0.07 (0) | 0.06 (0) |
|  | Mac0 | 2003 | 268 | 0.97 | 0.97 | 0.07 (0) | 0.07 (0) |
|  | Mac1 | 4867 | 658 | 0.91 | 0.9 | 0.07 (0) | 0.07 (0) |
|  | Mac2 | 3733 | 474 | 0.95 | 0.94 | 0.07 (0) | 0.06 (0) |
|  | MK | 2629 | 402 | 0.92 | 0.92 | 0.09 (0) | 0.07 (0) |
|  | Mon | 2483 | 330 | 0.91 | 0.9 | 0.08 (0) | 0.07 (0) |
|  | nCD4 | 2975 | 359 | 0.97 | 0.97 | 0.07 (0) | 0.06 (0) |
|  | nCD8 | 2774 | 339 | 0.93 | 0.93 | 0.07 (0) | 0.06 (0) |
|  | Neu | 4661 | 596 | 0.96 | 0.96 | 0.07 (0) | 0.06 (0) |
| FANTOM Gencode SPRITE cutoff 100 | GM12878 | 38 | 25 | 1 | 1 | 0 (0) | 0 (0) |
| FANTOM Gencode SPRITE cutoff 50 | GM12878 | 497 | 239 | 0.92 | 0.84 | 0.33 (0) | 0.2 (0) |
| FANTOM Gencode SPRITE cutoff 30 | GM12878 | 3381 | 1523 | 0.89 | 0.82 | 0.29 (0) | 0.2 (0) |
| ChromHMM Gencode SPRITE cutoff 100 | GM12878 | 622 | 94 | 0.96 | 0.95 | 0.02 (0) | 0.02 (0) |
| ChromHMM Gencode SPRITE cutoff 50 | GM12878 | 4794 | 663 | 0.85 | 0.84 | 0.06 (0) | 0.06 (0) |
| ChromHMM Gencode SPRITE cutoff 30 | GM12878 | 36027 | 4210 | 0.71 | 0.7 | 0.06 (0) | 0.05 (0) |

**Table S3:** Clusters of enhancers. Using the sharing enhancers (BCC > 0) we generated cluster of enhancers. An enhancer shares at least one promoter target with all the other enhancers in its cluster.

|  | Cell lines | Enhancers | Enhancer clusters | % of total enhancers in clusters | Average size of a cluster |
| --- | --- | --- | --- | --- | --- |
| FANTOM | GM12878 | 229 | 54 | 58.95 | 2.5 |
|  | HeLa | 10 | 1 | 40 | 4 |
|  | HMEC | 201 | 39 | 50.25 | 2.59 |
|  | HUVEC | 9 | 3 | 66.67 | 2 |
|  | IMR90 | 125 | 23 | 40.8 | 2.22 |
|  | K562 | 37 | 4 | 24.32 | 2.25 |
|  | KBM7 | 5 | 0 | 0 | NA |
|  | NHEK | 0 | 0 | 0 | NA |
| ChromHMM | GM12878 | 1914 | 420 | 93.83 | 4.28 |
|  | HeLa | 32 | 10 | 84.38 | 2.7 |
|  | HMEC | 1907 | 450 | 94.65 | 4.01 |
|  | HUVEC | 86 | 24 | 95.35 | 3.42 |
|  | IMR90 | 490 | 126 | 69.18 | 2.69 |
|  | K562 | 536 | 131 | 94.22 | 3.85 |
|  | NHEK | 0 | 0 | 0 | NA |
| FANTOM (cutoffs) | GM12878 | 783 | 136 | 42.27 | 2.43 |
|  | HMEC | 277 | 21 | 16.25 | 2.14 |
|  | HUVEC | 41 | 5 | 29.27 | 2.4 |
|  | IMR90 | 468 | 70 | 32.69 | 2.19 |
|  | K562 | 440 | 72 | 37.05 | 2.26 |
|  | KBM7 | 1308 | 135 | 22.63 | 2.19 |
|  | NHEK | 200 | 20 | 20.5 | 2.05 |
| ChromHMM (cutoffs) | GM12878 | 9343 | 2134 | 87.51 | 3.83 |
|  | HMEC | 3635 | 927 | 66.8 | 2.62 |
|  | HUVEC | 458 | 121 | 70.09 | 2.65 |
|  | IMR90 | 2118 | 546 | 67.99 | 2.64 |
|  | K562 | 6835 | 1639 | 86.06 | 3.59 |
|  | NHEK | 3028 | 805 | 72.09 | 2.71 |

Here we used the IEPs with FANTOM and ChromHMM enhancers and GENCODE promoters using Rao looplists and cutoffs (400 for GM12878 and 100 for other cell lines.)

**Table S4:** The distance between each consecutive enhancer pairs in an enhancer clusters are shown in the left columns of the table. The right columns show the distance between each consecutive target pairs of the targets of the enhancers in an enhancer cluster.

|  | Cell lines | Enhancer clusters | **Distance distribution between the enhancers in the same cluster** | | | | | | **Distance distribution between the targets of enhancers in the same cluster** | | | | | |
| --- | --- | --- | --- | --- | --- | --- | --- | --- | --- | --- | --- | --- | --- | --- |
|  |  |  | <=1kb | > 1kb and <= 5kb | > 5kb and <= 10kb | > 10kb and <= 50kb | > 50kb | Diff Chrom | <=1kb | > 1kb and <= 5kb | > 5kb and <= 10kb | > 10kb and <= 50kb | > 50kb | Diff Chrom |
| FANTOM | GM12878 | 54 | 52.99 | 36.15 | 0 | 2.47 | 8.4 | 0 | 62.96 | 11.11 | 0 | 0 | 25.93 | 0 |
|  | HeLa | 1 | 16.67 | 83.33 | 0 | 0 | 0 | 0 | NA | NA | NA | NA | NA | NA |
|  | HMEC | 39 | 49.57 | 34.02 | 0 | 5.81 | 10.6 | 0 | 64.29 | 14.29 | 0 | 0 | 21.43 | 0 |
|  | HUVEC | 3 | 33.33 | 66.67 | 0 | 0 | 0 | 0 | NA | NA | NA | NA | NA | NA |
|  | IMR90 | 23 | 77.54 | 13.77 | 0 | 0 | 8.7 | 0 | 66.67 | 0 | 0 | 0 | 33.33 | 0 |
|  | K562 | 4 | 50 | 25 | 0 | 25 | 0 | 0 | 100 | 0 | 0 | 0 | 0 | 0 |
|  | KBM7 | 0 | NA | NA | NA | NA | NA | NA | NA | NA | NA | NA | NA | NA |
|  | NHEK | 0 | NA | NA | NA | NA | NA | NA | NA | NA | NA | NA | NA | NA |
| ChromHMM | GM12878 | 420 | 61.25 | 30.78 | 0 | 0.63 | 7.34 | 0 | 60.16 | 16.51 | 0 | 1.98 | 21.35 | 0 |
|  | HeLa | 10 | 65.33 | 34.67 | 0 | 0 | 0 | 0 | 0 | 100 | 0 | 0 | 0 | 0 |
|  | HMEC | 450 | 67.21 | 28.88 | 0 | 0.81 | 3.1 | 0 | 70.57 | 17.76 | 0 | 1.02 | 10.65 | 0 |
|  | HUVEC | 24 | 77.64 | 22.36 | 0 | 0 | 0 | 0 | 33.33 | 66.67 | 0 | 0 | 0 | 0 |
|  | IMR90 | 126 | 71.12 | 22.83 | 0 | 0 | 6.05 | 0 | 71.43 | 21.43 | 0 | 0 | 7.14 | 0 |
|  | K562 | 131 | 70.77 | 26.69 | 0 | 0.22 | 2.32 | 0 | 77.78 | 18.52 | 0 | 3.7 | 0 | 0 |
|  | NHEK | 0 | NA | NA | NA | NA | NA | NA | NA | NA | NA | NA | NA | NA |
| FANTOM (cutoffs) | GM12878 | 136 | 46.06 | 34.33 | 8.33 | 10.54 | 0.74 | 0 | 77.08 | 14.58 | 2.08 | 6.25 | 0 | 0 |
|  | HMEC | 21 | 77.78 | 0 | 22.22 | 0 | 0 | 0 | 83.33 | 0 | 16.67 | 0 | 0 | 0 |
|  | HUVEC | 5 | 73.33 | 26.67 | 0 | 0 | 0 | 0 | 100 | 0 | 0 | 0 | 0 | 0 |
|  | IMR90 | 70 | 67.62 | 13.33 | 6.19 | 11.43 | 1.43 | 0 | 33.33 | 25.93 | 22.22 | 7.41 | 11.11 | 0 |
|  | K562 | 72 | 59.03 | 22.45 | 9.72 | 8.8 | 0 | 0 | 58.97 | 17.95 | 7.69 | 15.38 | 0 | 0 |
|  | KBM7 | 135 | 48.84 | 18.72 | 21.23 | 1.48 | 9.73 | 0 | 59.87 | 6 | 6.67 | 2.67 | 24.8 | 0 |
|  | NHEK | 20 | 76.67 | 0 | 18.33 | 0 | 5 | 0 | 100 | 0 | 0 | 0 | 0 | 0 |
| ChromHMM (cutoffs) | GM12878 | 2134 | 64.27 | 19.01 | 8.7 | 7.43 | 0.59 | 0 | 49.23 | 15.04 | 14.56 | 17.32 | 3.85 | 0 |
|  | HMEC | 927 | 86.13 | 3.03 | 9.56 | 0.22 | 1.05 | 0 | 61.04 | 4.57 | 20.25 | 2.7 | 11.44 | 0 |
|  | HUVEC | 121 | 84.58 | 6.87 | 4.35 | 0 | 4.2 | 0 | 87.18 | 0 | 0 | 0 | 12.82 | 0 |
|  | IMR90 | 546 | 81.92 | 7.32 | 3.18 | 6.13 | 1.46 | 0 | 50.97 | 11.67 | 7.98 | 25.02 | 4.36 | 0 |
|  | K562 | 1639 | 69.61 | 15.73 | 8 | 5.72 | 0.94 | 0 | 43.9 | 14.44 | 19.1 | 19.13 | 3.43 | 0 |
|  | NHEK | 805 | 85.01 | 3.51 | 9.82 | 0.3 | 1.36 | 0 | 69.21 | 3.35 | 17.48 | 1.94 | 8.02 | 0 |

Here we used the IEPs with FANTOM and ChromHMM enhancers and GENCODE promoters using Rao looplists and cutoffs (400 for GM12878 and 100 for other cell lines.)

**Table S5:** The average distance between each enhancer pairs in enhancer clusters and in randomly chosen enhancers that do not belong to any cluster. The difference between the distances in the two groups are shown in p-values which were measured using Mann-Whitney U test with the alternative hypothesis being “the distances in an enhancer cluster are smaller than the randomly chosen non-cluster enhancers”.

|  | Cell Lines | Distance (cluster) | Distance (non-cluster) | Distance difference (P-value) |
| --- | --- | --- | --- | --- |
| FANTOM | GM12878 | 37806 | 45756485 | 0.34 |
|  | HELA | 1736 | 39067658 | 0 |
|  | HMEC | 25622 | 59214548 | 0.28 |
|  | HUVEC | 1581 | 22565071 | 0.5 |
|  | IMR90 | 40078 | 50774410 | 0.44 |
|  | K562 | 11222 | 21003610 | 0.39 |
|  | KBM7 | NA | NA | NA |
|  | NHEK | NA | NA | NA |
| ChromHMM | GM12878 | 18547 | 48926756 | 0.14 |
|  | HELA | 1165 | 40711704 | 0.35 |
|  | HMEC | 6374 | 51533111 | 0.13 |
|  | HUVEC | 854 | 52275946 | 0.16 |
|  | IMR90 | 16817 | 52234764 | 0.32 |
|  | K562 | 2708 | 48992474 | 0.13 |
|  | NHEK | NA | NA | NA |
| FANTOM (cutoffs) | GM12878 | 9834 | 49467032 | 0.35 |
|  | HMEC | 2034 | 45604666 | 0.45 |
|  | HUVEC | 1256 | 71257127 | 0.4 |
|  | IMR90 | 632624 | 53976141 | 0.43 |
|  | K562 | 2564 | 53807126 | 0.4 |
|  | KBM7 | 2953682 | 51555371 | 0.44 |
|  | NHEK | 1126083 | 52425078 | 0.48 |
| ChromHMM (cutoffs) | GM12878 | 93505 | 46545996 | 0.18 |
|  | HMEC | 315205 | 48244423 | 0.32 |
|  | HUVEC | 501602 | 46804947 | 0.32 |
|  | IMR90 | 234070 | 48013578 | 0.3 |
|  | K562 | 159441 | 46609253 | 0.19 |
|  | NHEK | 428598 | 53430250 | 0.3 |

Although the average distance between enhancers in clusters is much smaller than that of pairs of random enhancers, the Mann-Whitney p-value is large because of the existence of large distances between enhancer pairs in the same clusters.

**Table S6:** Overlap between enhancer clusters and super-enhancers. The percentage of enhancer clusters overlapped with the super-enhancers and the percentage of super-enhancers overlapped with the enhancer clusters are shown. The right columns of the table show that, among the percentage of enhancer clusters overlapping with the super-enhancers (5th column), what percentage of enhancer clusters are involved in different amount of region overlap. This shows on average whether a cluster have a high or low region overlap with a super-enhancer.

|  | Cell lines | Number of clusters | Number of super enhancers | % of clusters overlapped with super enhancers | % of super enhancers overlapped with clusters | Distribution of the percentage of overlap between enhancer clusters and super enhancers | | | |
| --- | --- | --- | --- | --- | --- | --- | --- | --- | --- |
|  |  |  |  |  |  | <= 25% | > 25% and <= 50% | > 50% and <= 75% | > 75% and <= 100% |
| FANTOM | GM12878 | 54 | 257 | 25.93 | 5.45 | 5.56 | 3.7 | 0 | 16.67 |
|  | HeLa | 1 | 698 | 100 | 0.14 | 0 | 0 | 0 | 100 |
|  | HMEC | 39 | 1099 | 51.28 | 2.46 | 12.82 | 2.56 | 2.56 | 33.33 |
|  | HUVEC | 3 | 912 | 33.33 | 0.11 | 0 | 0 | 0 | 33.33 |
|  | IMR90 | 23 | 502 | 26.09 | 1.79 | 4.35 | 0 | 0 | 21.74 |
|  | K562 | 4 | 742 | 50 | 0.27 | 0 | 0 | 25 | 25 |
|  | KBM7 | 5 | NA | NA | NA | NA | NA | NA | NA |
|  | NHEK | 0 | 1024 | NA | NA | NA | NA | NA | NA |
| ChromHMM | GM12878 | 420 | 257 | 9.52 | 15.56 | 1.9 | 0.71 | 0.48 | 6.43 |
|  | HeLa | 10 | 698 | 40 | 0.57 | 0 | 0 | 0 | 40 |
|  | HMEC | 450 | 1099 | 21.56 | 9.37 | 2.67 | 0.89 | 1.56 | 16.44 |
|  | HUVEC | 24 | 912 | 33.33 | 0.88 | 0 | 0 | 0 | 33.33 |
|  | IMR90 | 126 | 502 | 15.08 | 4.98 | 4.76 | 1.59 | 0 | 8.73 |
|  | K562 | 131 | 742 | 20.61 | 3.64 | 1.53 | 0 | 3.82 | 15.27 |
|  | NHEK | 0 | 1024 | NA | NA | NA | NA | NA | NA |
| FANTOM (cutoffs) | GM12878 | 136 | 257 | 21.32 | 10.51 | 0 | 0 | 0.74 | 20.59 |
|  | HMEC | 21 | 1099 | 38.1 | 0.73 | 0 | 0 | 0 | 38.1 |
|  | HUVEC | 5 | 912 | 40 | 0.22 | 0 | 0 | 0 | 40 |
|  | IMR90 | 70 | 502 | 21.43 | 4.38 | 1.43 | 1.43 | 1.43 | 17.14 |
|  | K562 | 72 | 742 | 36.11 | 3.37 | 0 | 0 | 2.78 | 33.33 |
|  | KBM7 | 1308 | NA | NA | NA | NA | NA | NA | NA |
|  | NHEK | 20 | 1024 | 45 | 1.86 | 5 | 0 | 0 | 40 |
| ChromHMM (cutoffs) | GM12878 | 2134 | 257 | 7.31 | 48.64 | 0.98 | 0.28 | 0.7 | 5.34 |
|  | HMEC | 927 | 1099 | 18.02 | 22.57 | 2.37 | 0.11 | 0.65 | 14.89 |
|  | HUVEC | 121 | 912 | 19.01 | 4.71 | 4.13 | 0 | 1.65 | 13.22 |
|  | IMR90 | 546 | 502 | 6.41 | 17.13 | 1.47 | 0.37 | 0.37 | 4.21 |
|  | K562 | 1639 | 742 | 17.08 | 39.62 | 2.56 | 2.14 | 1.89 | 10.49 |
|  | NHEK | 805 | 1024 | 18.01 | 24.02 | 1.99 | 0.75 | 1.49 | 13.79 |

Here we used the IEPs with FANTOM and ChromHMM enhancers and GENCODE promoters using Rao looplists and cutoffs (400 for GM12878 and 100 for other cell lines.)

**Table S7:** Average percentage of enhancers in the same clusters mapped in a common TAD or TAD gap. We mapped the enhancer clusters to the defined TADs and the gaps between two TADs. On average, almost all of the enhancers in a cluster were found to be located within the same TAD or TAD gap.

|  | Cell lines | Enhancer clusters | Relevant enhancer clusters (having at least 2 enhancers assigned to tads) | % of relevant enhancer clusters belonging to common tad | Relevant enhancer clusters (having at least 2 enhancers assigned to tad gap) | % of relevant enhancer clusters belonging to common tad gap |
| --- | --- | --- | --- | --- | --- | --- |
| FANTOM | GM12878 | 54 | 52 | 98.08 | 11 | 100 |
|  | HeLa | 1 | 0 | 0 | 1 | 100 |
|  | HMEC | 39 | 24 | 100 | 17 | 94.12 |
|  | HUVEC | 3 | 2 | 100 | 1 | 100 |
|  | IMR90 | 23 | 22 | 100 | 4 | 100 |
|  | K562 | 4 | 3 | 100 | 1 | 100 |
|  | KBM7 | 0 | NA | NA | NA | NA |
|  | NHEK | 0 | NA | NA | NA | NA |
|  | IMR90 (Dixon) | 23 | 20 | 100 | 3 | 100 |
| ChromHMM | GM12878 | 420 | 401 | 98.75 | 112 | 98.21 |
|  | HeLa | 10 | 6 | 100 | 4 | 100 |
|  | HMEC | 450 | 295 | 100 | 180 | 99.44 |
|  | HUVEC | 24 | 20 | 100 | 7 | 100 |
|  | IMR90 | 126 | 114 | 100 | 24 | 100 |
|  | K562 | 131 | 99 | 100 | 26 | 100 |
|  | NHEK | 0 | NA | NA | NA | NA |
|  | IMR90 (Dixon) | 126 | 121 | 99.17 | 6 | 100 |
| FANTOM (cutoffs) | GM12878 | 136 | 121 | 100 | 26 | 100 |
|  | HMEC | 21 | 10 | 100 | 11 | 100 |
|  | HUVEC | 5 | 1 | 100 | 4 | 100 |
|  | IMR90 | 70 | 56 | 100 | 18 | 100 |
|  | K562 | 72 | 61 | 100 | 12 | 100 |
|  | KBM7 | 135 | 109 | 98.17 | 37 | 100 |
|  | NHEK | 20 | 10 | 100 | 11 | 100 |
|  | IMR90 (Dixon) | 70 | 64 | 100 | 6 | 100 |
| ChromHMM (cutoffs) | GM12878 | 2134 | 1814 | 99.12 | 616 | 99.35 |
|  | HMEC | 927 | 517 | 100 | 445 | 99.33 |
|  | HUVEC | 121 | 65 | 98.46 | 62 | 95.16 |
|  | IMR90 | 546 | 435 | 99.31 | 142 | 100 |
|  | K562 | 1639 | 1196 | 99.16 | 526 | 99.24 |
|  | NHEK | 805 | 450 | 99.33 | 389 | 99.49 |
|  | IMR90 (Dixon) | 546 | 485 | 99.18 | 63 | 100 |

Here we used the IEPs with FANTOM and ChromHMM enhancers and GENCODE promoters using Rao looplists and cutoffs (400 for GM12878 and 100 for other cell lines.)

**Table S8:** Percentages of common enhancer clusters between two cell lines.

|  | Cell line1 | Cell line2 | Clusters in cell line 1 | Clusters in cell line 2 | % of common clusters (with respect to cell line 1) | % of common clusters (with respect to cell line 2) |
| --- | --- | --- | --- | --- | --- | --- |
| FANTOM | GM12878 | HeLa | 54 | 1 | 0 | 0 |
|  | GM12878 | HMEC | 54 | 39 | 1.85 | 2.56 |
|  | GM12878 | HUVEC | 54 | 3 | 0 | 0 |
|  | GM12878 | IMR90 | 54 | 23 | 3.7 | 8.7 |
|  | GM12878 | K562 | 54 | 4 | 3.7 | 50 |
|  | GM12878 | KBM7 | 54 | 0 | NA | NA |
|  | GM12878 | NHEK | 54 | 0 | NA | NA |
|  | HeLa | HMEC | 1 | 39 | 100 | 2.56 |
|  | HeLa | HUVEC | 1 | 3 | 0 | 0 |
|  | HeLa | IMR90 | 1 | 23 | 0 | 0 |
|  | HeLa | K562 | 1 | 4 | 0 | 0 |
|  | HeLa | KBM7 | 1 | 0 | NA | NA |
|  | HeLa | NHEK | 1 | 0 | NA | NA |
|  | HMEC | HUVEC | 39 | 3 | 0 | 0 |
|  | HMEC | IMR90 | 39 | 23 | 2.56 | 4.35 |
|  | HMEC | K562 | 39 | 4 | 0 | 0 |
|  | HMEC | KBM7 | 39 | 0 | NA | NA |
|  | HMEC | NHEK | 39 | 0 | NA | NA |
|  | HUVEC | IMR90 | 3 | 23 | 0 | 0 |
|  | HUVEC | K562 | 3 | 4 | 0 | 0 |
|  | HUVEC | KBM7 | 3 | 0 | NA | NA |
|  | HUVEC | NHEK | 3 | 0 | NA | NA |
|  | IMR90 | K562 | 23 | 4 | 4.35 | 25 |
|  | IMR90 | KBM7 | 23 | 0 | NA | NA |
|  | IMR90 | NHEK | 23 | 0 | NA | NA |
|  | K562 | KBM7 | 4 | 0 | NA | NA |
|  | K562 | NHEK | 4 | 0 | NA | NA |
|  | KBM7 | NHEK | 0 | 0 | NA | NA |
| ChromHMM | GM12878 | HeLa | 420 | 10 | 0 | 0 |
|  | GM12878 | HMEC | 420 | 450 | 1.43 | 1.33 |
|  | GM12878 | HUVEC | 420 | 24 | 0.24 | 4.17 |
|  | GM12878 | IMR90 | 420 | 126 | 0.71 | 2.38 |
|  | GM12878 | K562 | 420 | 131 | 0.71 | 2.29 |
|  | GM12878 | KBM7 | 420 | 0 | NA | NA |
|  | HeLa | HMEC | 10 | 450 | 10 | 0.22 |
|  | HeLa | HUVEC | 10 | 24 | 0 | 0 |
|  | HeLa | IMR90 | 10 | 126 | 0 | 0 |
|  | HeLa | K562 | 10 | 131 | 10 | 0.76 |
|  | HeLa | KBM7 | 10 | 0 | NA | NA |
|  | HMEC | HUVEC | 450 | 24 | 0.22 | 4.17 |
|  | HMEC | IMR90 | 450 | 126 | 0.67 | 2.38 |
|  | HMEC | K562 | 450 | 131 | 0.89 | 3.05 |
|  | HMEC | KBM7 | 450 | 0 | NA | NA |
|  | HUVEC | IMR90 | 24 | 126 | 4.17 | 0.79 |
|  | HUVEC | K562 | 24 | 131 | 4.17 | 0.76 |
|  | HUVEC | KBM7 | 24 | 0 | NA | NA |
|  | IMR90 | K562 | 126 | 131 | 0.79 | 0.76 |
|  | IMR90 | KBM7 | 126 | 0 | NA | NA |
|  | K562 | KBM7 | 131 | 0 | NA | NA |
| FANTOM (cutoffs) | GM12878 | HeLa | 136 | 21 | 6.62 | 42.86 |
|  | GM12878 | HMEC | 136 | 5 | 1.47 | 40 |
|  | GM12878 | HUVEC | 136 | 70 | 14.71 | 28.57 |
|  | GM12878 | IMR90 | 136 | 72 | 28.68 | 54.17 |
|  | GM12878 | K562 | 136 | 135 | 14.71 | 14.81 |
|  | GM12878 | KBM7 | 136 | 20 | 7.35 | 50 |
|  | HeLa | HMEC | 21 | 5 | 9.52 | 40 |
|  | HeLa | HUVEC | 21 | 70 | 42.86 | 12.86 |
|  | HeLa | IMR90 | 21 | 72 | 47.62 | 13.89 |
|  | HeLa | K562 | 21 | 135 | 100 | 15.56 |
|  | HeLa | KBM7 | 21 | 20 | 52.38 | 55 |
|  | HMEC | HUVEC | 5 | 70 | 40 | 2.86 |
|  | HMEC | IMR90 | 5 | 72 | 40 | 2.78 |
|  | HMEC | K562 | 5 | 135 | 80 | 2.96 |
|  | HMEC | KBM7 | 5 | 20 | 40 | 10 |
|  | HUVEC | IMR90 | 70 | 72 | 22.86 | 22.22 |
|  | HUVEC | K562 | 70 | 135 | 11.43 | 5.93 |
|  | HUVEC | KBM7 | 70 | 20 | 5.71 | 20 |
|  | IMR90 | K562 | 72 | 135 | 26.39 | 14.07 |
|  | IMR90 | KBM7 | 72 | 20 | 15.28 | 55 |
|  | K562 | KBM7 | 135 | 20 | 13.33 | 90 |
| ChromHMM (cutoffs) | GM12878 | HeLa | 2755 | 386 | 0.58 | 4.15 |
|  | GM12878 | HMEC | 2755 | 112 | 0.18 | 4.46 |
|  | GM12878 | HUVEC | 2755 | 1031 | 0.83 | 2.23 |
|  | GM12878 | IMR90 | 2755 | 2076 | 4.07 | 5.11 |
|  | GM12878 | K562 | 2755 | 1433 | 1.71 | 3.21 |
|  | HeLa | HMEC | 386 | 112 | 1.81 | 6.25 |
|  | HeLa | HUVEC | 386 | 1031 | 2.85 | 1.07 |
|  | HeLa | IMR90 | 386 | 2076 | 6.99 | 1.35 |
|  | HeLa | K562 | 386 | 1433 | 6.48 | 1.74 |
|  | HMEC | HUVEC | 112 | 1031 | 6.25 | 0.68 |
|  | HMEC | IMR90 | 112 | 2076 | 9.82 | 0.63 |
|  | HMEC | K562 | 112 | 1433 | 5.36 | 0.42 |
|  | HUVEC | IMR90 | 1031 | 2076 | 3.1 | 1.59 |
|  | HUVEC | K562 | 1031 | 1433 | 3.2 | 2.23 |
|  | IMR90 | K562 | 2076 | 1433 | 2.75 | 3.98 |

Here we used the IEPs with FANTOM and ChromHMM enhancers and GENCODE promoters using Rao looplists and cutoffs (400 for GM12878 and 100 for other cell lines.)

**Table S9:** Gene ontology enrichment analysis for the target genes of the enhancer clusters.

|  | Cell lines | # Cluster targets | # GO Terms associated with cluster targets | Most significant GO Terms (p-value < 1e-05) |
| --- | --- | --- | --- | --- |
| FANTOM | GM12878 | 73 | 4 | Protease binding, Nucleoplasm, DNA binding |
|  | HELA | 1 | 0 |  |
|  | HMEC | 51 | 0 |  |
|  | HUVEC | 3 | 0 |  |
|  | IMR90 | 27 | 0 |  |
|  | K562 | 7 | 3 | Nucleosome, DNA packaging complex |
|  | KBM7 | 0 | 0 |  |
|  | NHEK | 0 | 0 |  |
| ChromHMM | GM12878 | 521 | 45 | Nuclear nucleosome, Chromatin silencing at rDNA, Nucleosome, DNA packaging complex, Chromatin silencing |
|  | HELA | 11 | 0 |  |
|  | HMEC | 598 | 1 |  |
|  | HUVEC | 27 | 0 |  |
|  | IMR90 | 141 | 21 | Negative regulation of G1/S transition of mitotic cell cycle, Negative regulation of cell cycle G1/S phase transition, DNA damage response, signal transduction by p53 class mediator resulting in cell cycle arrest, Signal transduction involved in mitotic G1 DNA damage checkpoint, Signal transduction involved in DNA damage checkpoint |
|  | K562 | 159 | 0 |  |
|  | NHEK | 0 | 0 |  |
| FANTOM (cutoffs) | GM12878 | 157 | 2 | Immune response |
|  | HMEC | 26 | 0 |  |
|  | HUVEC | 6 | 0 |  |
|  | IMR90 | 80 | 0 |  |
|  | K562 | 86 | 0 |  |
|  | KBM7 | 168 | 5 | Immune response, Regulation of DNA demethylation, Innate immune response, Defense response |
|  | NHEK | 22 | 0 |  |
| ChromHMM (cutoffs) | GM12878 | 2619 | 165 | Nucleosome, Chromatin silencing, DNA packaging complex, Chromatin silencing at rDNA, DNA replication-dependent nucleosome assembly |
|  | HMEC | 1110 | 10 | MLL1 complex, Melanosome, Protein heterotetramerization, Clathrin-coated vesicle membrane, Cell junction assembly |
|  | HUVEC | 136 | 0 |  |
|  | IMR90 | 635 | 0 |  |
|  | K562 | 1971 | 44 | NcRNA metabolic process, NcRNA processing, Chromatin silencing, RRNA metabolic process, MRNA catabolic process |
|  | NHEK | 898 | 49 | Chromatin silencing at rDNA, DNA replication-dependent nucleosome assembly, Positive regulation of gene expression, epigenetic, Negative regulation of megakaryocyte differentiation, Regulation of gene silencing by miRNA |

**Table S10:** The average sequence similarity score among the enhancers in a cluster and in randomly chosen enhancers that do not belong to any cluster. The difference between the sequence similarity scores between the two groups are shown in p-values which were measured using Mann-Whitney U test with the alternative hypothesis being “the sequence similarity in an enhancer cluster are different than the randomly chosen non-cluster enhancers”.

|  | Cell Lines | Sequence similarity score (cluster) | Sequence similarity score (random) | Difference in sequence similarity (P-Value) |
| --- | --- | --- | --- | --- |
| FANTOM | GM12878 | 0.08 | 0.08 | 0.64 |
|  | HELA | 0.02 | 0.02 | 0.89 |
|  | HMEC | 0.08 | 0.07 | 0.55 |
|  | HUVEC | 0.08 | 0.07 | 0.4 |
|  | IMR90 | 0.09 | 0.09 | 0.59 |
|  | K562 | 0.12 | 0.11 | 0.69 |
|  | KBM7 | NA | NA | NA |
|  | NHEK | NA | NA | NA |
| ChromHMM | GM12878 | 0.04 | 0.03 | 0.47 |
|  | HELA | 0.05 | 0.07 | 0.47 |
|  | HMEC | 0.03 | 0.03 | 0.5 |
|  | HUVEC | 0.03 | 0.04 | 0.4 |
|  | IMR90 | 0.07 | 0.07 | 0.46 |
|  | K562 | 0.04 | 0.03 | 0.5 |
|  | NHEK | NA | NA | NA |
| FANTOM (cutoffs) | GM12878 | 0.08 | 0.08 | 0.57 |
|  | HMEC | 0.08 | 0.09 | 0.52 |
|  | HUVEC | 0.06 | 0.07 | 0.53 |
|  | IMR90 | 0.09 | 0.08 | 0.54 |
|  | K562 | 0.09 | 0.09 | 0.54 |
|  | KBM7 | 0.1 | 0.1 | 0.56 |
|  | NHEK | 0.09 | 0.09 | 0.5 |
| ChromHMM (cutoffs) | GM12878 | 0.04 | 0.04 | 0.49 |
|  | HMEC | 0.06 | 0.05 | 0.53 |
|  | HUVEC | 0.06 | 0.05 | 0.51 |
|  | IMR90 | 0.07 | 0.07 | 0.47 |
|  | K562 | 0.04 | 0.04 | 0.5 |
|  | NHEK | 0.06 | 0.06 | 0.53 |
